# Acknowledgement of Reviewers 2021

**DOI:** 10.3389/phrs.2022.1605136

**Published:** 2022-09-01

**Authors:** PHR Editorial Office

**Affiliations:** Swiss School of Public Health (SSPH+), Zürich, Switzerland

The Editors of Public Health Reviews would like to thank all Reviewers in 2021 for the time they have spent and the valuable contributions they have made to ensure the scientific quality of the journal.

Christine A’Court, United KingdomBabatunde Akinwunmi, United States Sinaa Al Aqeel, Saudi ArabiaChantal Arditi, SwitzerlandHélène Aschmann, United StatesInês Baía, PortugalEduard Baladia, SpainRebecca Bennett, AustraliaTomasz Bochenek, PolandKatarina Braun, United States Katie Cederberg, United StatesHarrison Ng Chok, AustraliaAna Cruz, PortugalNicole Geovana Dias, BrazilDominik Dietler, SwitzerlandNicola Diviani, SwitzerlandAlicja Domagała, PolandMarta Fadda, SwitzerlandGuihong Fan, United StatesMichele Dalla Fontana, BrazilSaswata Ghosh, IndiaGabriel Gulis, DenmarkLouise Hartley, United KingdomHerwansyah Herwansyah, NetherlandsMusa Abubakar Kana, NigeriaMelkamu Dugassa Kassa, South AfricaRobin Van Kessel, NetherlandsAnisur Khan, BangladeshAndrew Kim, United StatesEvdoxia Kyriazopoulou, GreeceRaquel Lucas, PortugalTim Lucas, United KingdomLaura Magaña, United StatesPatricia McDaniel, United StatesSinenhlanhla Memela, South AfricaGage Moreno, United StatesAlexandra Moura, FranceJessica Neicun, NetherlandsTaco Niet, CanadaRachel Nugent, United StatesEkwaro Obuku, UgandaOsorio, Camilo Gutierrez Osorio, ColombiaMadhuri Pattamatta, IndiaPaul, Ayan Paul, GermanyPaul Pavli, AustraliaPablo Perel, United KingdomPriyanka Priyanka, United StatesAna Catarina Queiroga, PortugalAzizur Rahman, AustraliaNandini Ramanujam, CanadaAndres Roman-Urrestarazu, United KingdomJoão Cavaleiro Rufo, PortugalMarco Scherz, AustriaDenise Sharon, United StatesB. Shayak, United StatesBritt Singletary, United StatesNanny Natalia Mulyani Soetedjo, IndonesiaAsha Soletti, NetherlandsMindaugas Stankūnas, LithuaniaAmbra Stefani, AustriaLiz Tobin-Tyler, United StatesSusanne Unverzagt, GermanyCherian Varghese, SwitzerlandDuoquan Wang, ChinaBrian Li Han Wong, SwitzerlandClaudia Zanini, SwitzerlandJan Anton Van Zanten, Netherlands

